# Newly discovered neuron-to-glioma communication: new noninvasive therapeutic opportunities on the horizon?

**DOI:** 10.1093/noajnl/vdab018

**Published:** 2021-02-04

**Authors:** Giulia Sprugnoli, Alexandra J Golby, Emiliano Santarnecchi

**Affiliations:** 1 Berenson-Allen Center for Noninvasive Brain Stimulation, Beth Israel Deaconess Medical Center, Harvard Medical School, Boston, Massachusetts, USA; 2 Radiology Unit, Department of Medicine and Surgery, University of Parma, Parma, Italy; 3 Departments of Neurosurgery and Radiology, Brigham and Women’s Hospital, Harvard Medical School, Boston, Massachusetts, USA

**Keywords:** gliomas, NiBS, noninvasive brain stimulation, plasticity, synapses

## Abstract

The newly discovered functional integration of glioma cells into brain networks in mouse models provides groundbreaking insight into glioma aggressiveness and resistance to treatments, also suggesting novel potential therapeutic avenues and targets. In the context of such neuron-to-glioma communication, noninvasive brain modulation techniques traditionally applied to modulate neuronal function in neurological and psychiatric diseases (eg, increase/decrease cortical excitability and plasticity) could now be tested in patients with brain tumors to suppress glioma’s activity and its pathological crosstalk with healthy brain tissue.

Key Points

• Gliomas have been found to be electrically active and integrate into the brain’s neural network.
• Noninvasive brain neuromodulation safely suppressed or enhances the neuronal activity in humans.
• Neuromodulation techniques may interfere with this neuron-to-glioma electrical communication.

Gliomas have recently been found to contain electrically active tissue integrated into the brain’s neural network.^1,2^ According to these seminal studies, gliomas are able to create electrical synapses with surrounding neural tissue and this communication drives tumor cell growth and migration, closing the circle with previously discovered influence of neuronal activity in regulating cancer growth.^3^ This groundbreaking novel molecular and pathophysiological framework could not only shed light on cancer proliferation and migration pathways, but also provide novel therapeutic opportunities aimed at inhibiting tumor-promoting neural activity. In this regard, noninvasive brain stimulation (NiBS) represents a group of techniques widely used in clinical and research settings to modify neuronal activity by means of transcranial magnetic or electrical fields applied on the scalp. Depending on shape, frequency, and intensity of stimulation, NiBS can lead to neuronal firing as in the case of transcranial magnetic stimulation (TMS), or cause an increase or decrease of membrane excitability as with transcranial direct current stimulation (tDCS). Interestingly, both TMS and tDCS can induce long-lasting effects especially when applied over multiple sessions, thanks to the modulation of synaptic plasticity and excitation/inhibition balance in the targeted regions^4^ (Figure 1A). Given their safety and efficacy, NiBS techniques—in particular TMS—, are FDA-approved for the treatment of conditions like medication-resistant depression, obsessive compulsive disorder (OCD), and migraine.^4^ NiBS has been extensively and successfully applied in both neurology and psychiatry; however, its potential use in patients with brain cancers has not been explored beyond pre-surgical functional mapping. To date, no clinical trials have been conducted on brain tumor patients via TMS or tDCS, probably due to the lack of knowledge about noninvasive brain stimulation in the field of clinical neuro-oncology. Indeed, neuromodulation techniques have been developed and tested mainly by neurologists and psychiatrists given the possibility of interacting with neuronal activity and potentially restoring physiological function,^5,6^ with extra-neuronal applications only now emerging (ie, brain perfusion modulation^7^). Moreover, as for the application of neuromodulation to inhibit neuronal activity induced glioma growth, new insight on tumor electrical property supporting a new context for NiBS application has been shared very recently, and still need to be fully received by the clinical neuro-oncological community. The discovery of this “*electric*” behavior in glioma cells opens intriguing scenarios, including whether NiBS could be used to interrupt the bidirectional signaling between healthy neuronal populations and cancer cells, therefore potentially slowing cancer growth and increasing patient survival.

**Figure 1.**
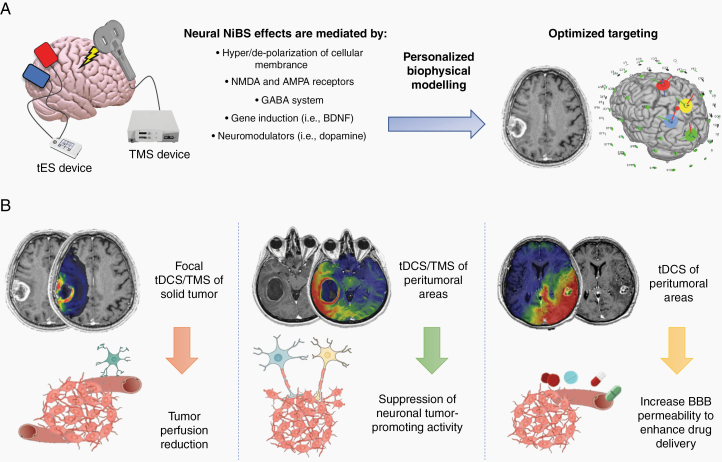
NiBS mechanisms of action and biological substrates for brain tumor applications. (A) TMS and tDCS devices are applied over the scalp, with a small amount of gel under the electrodes in the case of tDCS to facilitate electrical conductivity. Neural effects of NiBS are related to its action on multiple substrates, ranging from polarization of cellular membranes to gene induction, resulting in acute transitory effects as well as long-term plastic modifications after repeated stimulation sessions. Accurate and personalized biophysical modeling of current distribution on the basis of individual MRI is warranted to optimize targeting and to reduce the possibility of side effects. (B) NiBS can be delivered focally in the lesion to reduce tumor perfusion, on peritumoral areas to inhibit neuron-to-glioma communication that promotes tumor growth and invasiveness, as well as on the tumor and peritumoral regions to enhance drug delivery via an increase of BBB permeability. AMPA, α-amino-3-hydroxy-5-methyl-4-isoxazolepropionic acid; BBB, blood–brain barrier; BDNF, brain-derived neurotrophic factor; GABA, gamma-aminobutyric acid; NMDA, N-methyl-D-aspartate; tDCS, transcranial direct current stimulation; TMS, transcranial magnetic stimulation.

## Disrupting Neuron-to-Glioma Communication

While the importance of tumor microenvironment in regulating glioma progression is well established, the dependence of gliomas on neuronal activity has been only recently demonstrated, with data showing how neuronal spiking promotes glioma growth and proliferation in vivo.^1,2^ The *neuronal activity-regulated cancer growth* acts via a specific pathway involving the synaptic protein neuroligin-3 (NLGN3), whose expression inversely correlates with the survival of patients with glioblastoma.^3^ Interestingly, NLGN3 was found to induce the expression of numerous synaptic genes in glioma cells, leading the investigators to explore whether glioma cells could also engage in synaptic connections with surrounding neuronal populations. Surprisingly, excitatory synaptic structures between neurons and glioma cells (diffuse and anaplastic astrocytomas, glioblastomas, and diffuse intrinsic pontine gliomas) were observed by two independent groups in the United States and Germany,^1,2^ who further revealed how these specific synapses shared the same molecular properties of a classical type of neuron-to-neuron synapses involving calcium-permeable AMPA (ionotropic glutamate) receptors. Electrical stimulation of neurons surroundings glioma cells implanted in mouse models elicited a rapid depolarization in tumor cells displaying such specific synaptic structures. A long-lasting depolarizing current was also observed in a glioma subpopulation, which even more surprisingly spreads through the entire glioma network via gap junctions. The depolarizing current caused a calcium-ion influx that ultimately promoted cancer cell migration and mitosis. These growth-supporting changes were strongly inhibited by pharmacological and genetic blockage of the glutamate synaptic transmission.^1,2^

On the other hand, neuron-to-glioma communication does not seem unidirectional. Gliomas, in fact, can increase neuronal spiking via numerous mechanisms, such as synaptogenic factors, glutamate secretion, as well as by reducing the number of inhibitory interneurons in its microenvironment.^1^ To further shed light on this relation, Monje and colleagues explored fast oscillatory activity in the gamma frequency band (that correlates with neuronal spiking) in patients with high-grade gliomas (HGGs) via intraoperative electrocorticography, finding increased high-gamma spectral power in infiltrated brain tissue with respect to macroscopically normal-appearing tissue.^1^

In summary, HGGs seem to be tightly integrated in the brain electrical network and further able to create a positive feedback loop with healthy neurons to promote spiking activity, and thus tumor viability. These recent findings are also in line with the evidence of hyperexcitability in glioma patients that frequently leads to seizures, as well as with the observation that progression of epilepsy, when present, is associated to progression of tumor.^8^ However, further research is needed to disentangle the mechanisms of tumor-induced neuronal hyperexcitability, considering that low-grade glioma (LGG)—and not HGGs, are more prone to cause epilepsy.^9^

Even if effective modulation of “glioma cells’ excitability” has not been tested via NiBS yet and is difficult to foresee at the present time, NiBS application on surroundings neuronal tissue to modulate excitability and decrease the mitotic stimuli induced in the tumor cells represents a feasible target for future research. Indeed, NiBS acts on multiple levels to induce long-term depression-like effects (ie, indicating a decrease of synapses’ efficacy^10^), such as glutamatergic and calcium transmission, gene induction,^11^ brain-derived neurotrophic factor (BDNF)-dependent plasticity and modulation of multiple neurotransmitters^12,13^ (Figure 1A). Therefore, NiBS may represent a viable therapeutic strategy when considering the positive feedback on neural activity induced by glioma cells (Figure 1B). As anticipated, TMS can directly induce neuronal spiking via electromagnetic induction, as well as suppress action potentials in humans.^4^ The application of repeated magnetic stimuli (repetitive TMS – rTMS) over a cortical site using so-called “low-frequency” rTMS protocols (<1Hz) usually causes a long-lasting decrease in cortical activity, whereas high-frequency stimulation (eg, 10 Hz or 20 Hz) is commonly used to increase local excitability for various purposes, for example, boost cognitive function or modulate network connectivity.^4^ Recent evidence suggests a definite antidepressant efficacy of high-frequency rTMS over the left DLPFC.^14^ The rationale of NiBS application in depressed patients stems from the documented imbalance of neuronal activity between the bilateral DLPFC areas.^15,16^ In the case of drug-resistant OCD, hyperactivation of the supplementary motor area and/or other components of the cortico-striato-thalamo-cortical circuits is related to patient symptoms, and the application of inhibitory rTMS has been shown to ameliorate the symptoms for at least 6–12 weeks.^17,18^ Finally, in migraine with aura, single-pulse TMS is delivered to the occipital cortex at the beginning of symptomatology to interrupt the attack via inhibition of cortical spreading depression.^19^ When applied over time (eg, 3000 pulses over ~30 min daily for 6 weeks, as in the case of FDA-approved TMS therapy for treatment-resistant depression), TMS is able to induce long-lasting changes in brain connectivity and even affect structural brain properties.^4,20^

So far, TMS has been used only in the preoperative assessment of patients with brain tumors, to map eloquent cortical regions (eg, motor and language areas) by transiently exciting or suppressing their activity and thus guide surgical resection.^4^ Indeed, gliomas often involve—or are located near—eloquent areas, thus careful functional mapping is needed to optimize maximal safe tumor resection and improve survival. Single-pulse TMS is applied to the motor cortex to elicit a motor evoked potential (MEP) in a target muscle recorded via electromyography.^21^ For language mapping, rTMS is delivered to the language network while participants perform a language task to transiently disrupt speech or other language functions and identify areas to be spared during surgery. Navigated TMS enormously increases the accuracy of TMS for surgical mapping, with its performance being equivalent to the gold standard represented by Direct Cortical Stimulation performed intraoperatively.^21^ This has led to a consensus about the use of navigated TMS for routine neurosurgical work-up, with identification of standardized protocols.^21^ Different TMS protocols have led to various TMS performance in terms of functional mapping across studies.^22^ Importantly, lesions themselves can influence the performance of TMS for surgical mapping, due to location (ie, direct involvement of the eloquent regions) and type of tumor, considering that the typically slow LGG growth allows for functional plasticity and cortical rearrangement induced by neuronal damage. These changes are not often seen in HGG.^22^

In the light of recent new discoveries, the possibility to noninvasively inhibit neuronal activity and consequent mitogenic signaling in brain regions surrounding glioma cells is an intriguing possibility, with potential cascade effects on tumor growth and survival. Neuronavigated, image-guided inhibitory TMS (eg, continuous theta burst or low-frequency TMS) could be tested to exert fine control of neuronal activity in regions surroundings the glioma (leveraging TMS ~1 cm^3^ spatial resolution), transiently suppressing local neural activity (Figure 1B). Moreover, response to TMS as measured via electrophysiology could also represent a novel biomarker of tissue “health” (so-called Perturbation-Based Biomarkers), based on individual deviation from patterns of connectivity and excitability observed after stimulation in the healthy brain.^23^

On the other hand, tDCS, a recently developed form of NiBS, allows to deliver direct electrical current into the brain via scalp electrodes, with documented effects similar to those of TMS.^4^ The electrical field induced by tDCS is typically more widespread than the one induced by TMS and allows to target multiple brain regions at the same time via multiple scalp electrodes. Importantly, both TMS and tDCS have been shown to induce effects on cortical activity and connectivity with minimal scalp sensation, and even after a single short session of approximately 30 min. This is even more valuable in comparison to the collateral effects usually induced by the administration of a pharmacological drug that can interfere with neuron-to-glioma communication and slow glioma progression, as in the case of the AMPA-R antagonist Perampanel^1^ that widely inhibits AMPA-R in the brain, without however causing a selective action on the peritumoral regions.^24^ Moreover, portability and ease to use are also notable characteristics of tDCS, allowing for its safe administration in home environments^25^ with minimal burden for patients compared to, for example, recently approved NovoTTF for glioblastoma treatment (requiring up to 18 h stimulation per day).^26^ Differently from tDCS, TTF delivers alternating currents that oscillate at extraphysiological frequencies (eg, 200 kHz), therefore not affecting neuronal activity but rather impeding cancer cell mitosis by interfering with the formation of microtubules, making it not suitable for selective disruption of neuron-to-glioma communication. In this scenario, neuroimaging techniques (eg, functional MRI, perfusion MRI, positron emission tomography) could be used to identify regions that would benefit from the application of inhibitory TMS/tDCS, for example, peri-tumoral regions displaying the strongest link with HGGs. More ambitiously, prediction of tumor spread based on imaging and electrophysiology data could be used to map migration trajectories and potentially prevent tumor expansion by suppressing distant regions at higher probability of tumor migration.

On top of modulating neuronal and tumor’s electrical activity, noninvasive approaches to selectively modulate perfusion of intra and peritumoral regions could be complementary to those acting on tumor’s electrical behavior (Figure 1B). Gliomas perfusion is positively correlated with the World Health Organization (WHO) grade and negatively with survival.^27^ Image-guided, personalized tDCS targeting the solid tumor mass has been recently demonstrated able to transiently modify tumor perfusion in patients with glioblastoma and lung metastasis by our group, both pre- and postsurgery.^7^ Based on similar evidence on bodily tumors (eg, spinal tumor, breast, and liver cancers^28,29^) in which progressive perfusion reduction and necrosis have been observed after repetitive sessions of direct current stimulation delivered via electrodes directly inserted into the tumor, tDCS in gliomas may lead to long-lasting reduction of tumor perfusion and be leveraged to reduce tumor metabolism as well as growth.^28,29^ tDCS has also been shown to transiently increase permeability of the blood–brain barrier (BBB) to small and large molecules (up to 70 kDa) in rats and endothelial monolayers, using stimulation intensities similar to those applied in humans (1 mA).^30^ Enhancement/control of drug delivery into the brain (and specifically into the tumor) constitutes one of the major challenges in modern neuro-oncology, with combined tDCS-chemotherapy potentially offering a viable synergy for enhancing local drug delivery by means of modulation of BBB and perfusion in peritumoral regions (eg, 192 Da, Temozolomide).

Of note, currently available NiBS solutions could be immediately used in the context of clinical trials to explore the therapeutic potential of brain stimulation on brain tumors. As shown in a pilot study aimed at decreasing tumor perfusion,^16^ tDCS application in patients with tumors is safe and feasible, but personalized biophysical models need to be created in order to realistically estimate current diffusion, as well as to effectively target the lesion and/or the peritumoral areas and reduce the risk for potential collateral effects (Figure 1). Therefore, a potential limit of NiBS is represented by its operator dependency, including the need for careful biophysical modeling and personalized stimulation montage.^31^ Also, past literature shows individual variability in responsiveness to NiBS, with many biological and anatomical factors potentially determining the efficacy of transcranial neuromodulation.^18^ This should be expected in patients with brain tumor as well, where additional heterogeneity is contributed by the lesion itself and its molecular and biological characteristics. Finally, long-term neuromodulatory effects of NiBS should be explored in relation to the speed of tumor progression. Indeed, synaptic changes and plasticity rearrangements observed under repetitive treatment (ie, rTMS) usually leads to long-term modulation of neuronal activity in neuropsychiatric populations.^4,17,18,20^ Along with the strong reduction of proliferation observed in mice models after inhibition of neuron-to-glioma communication,^1,2^ a reduction in the amount of tumor growth specifically caused by this mechanism could also be expected (corresponding to ~50% decrease in proliferation^1,2^) when accurate modeling and repeated neuromodulatory sessions are administered.

Overall, growing evidence suggests an opportunity to investigate NiBS as a potential resource to slow down glioma progression, via suppression of tumor-promoting neuronal activity, modulation of perfusion, and enhanced drug permeability. Given the favorable safety profile, efficacy, noninvasiveness, and portability of TMS/tDCS devices, a conceptual framework for NiBS application in brain cancers should be thoroughly discussed and tested.

**Conflict of interest statement**. All authors report no conﬂict of interest.
